# Crystallization, dehydration and experimental phasing of WbdD, a bifunctional kinase and methyltransferase from *Escherichia coli* O9a

**DOI:** 10.1107/S0907444912029599

**Published:** 2012-09-18

**Authors:** Gregor Hagelueken, Hexian Huang, Karl Harlos, Bradley R. Clarke, Chris Whitfield, James H. Naismith

**Affiliations:** a Biomedical Sciences Research Complex, The University of St Andrews, North Haugh, St Andrews KY16 9ST, Scotland; b Division of Structural Biology, Wellcome Trust Centre for Human Genetics, University of Oxford, Roosevelt Drive, Oxford OX3 7BN, England; c Department of Molecular and Cellular Biology, University of Guelph, Ontario N1G 2W1, Canada

**Keywords:** WbdD, crystal dehydration

## Abstract

WbdD is a bifunctional kinase/methyltransferase that is responsible for regulation of lipopolysaccharide O antigen polysaccharide chain length in *Escherichia coli* serotype O9a. Solving the crystal structure of this protein proved to be a challenge because the available crystals belonging to space group *I*23 only diffracted to low resolution (>95% of the crystals diffracted to resolution lower than 4 Å and most only to 8 Å) and were non-isomorphous, with changes in unit-cell dimensions of greater than 10%. Data from a serendipitously found single native crystal that diffracted to 3.0 Å resolution were non-isomorphous with a lower (3.5 Å) resolution selenomethionine data set. Here, a strategy for improving poor (3.5 Å resolution) initial phases by density modification and cross-crystal averaging with an additional 4.2 Å resolution data set to build a crude model of WbdD is desribed. Using this crude model as a mask to cut out the 3.5 Å resolution electron density yielded a successful molecular-replacement solution of the 3.0 Å resolution data set. The resulting map was used to build a complete model of WbdD. The hydration status of individual crystals appears to underpin the variable diffraction quality of WbdD crystals. After the initial structure had been solved, methods to control the hydration status of WbdD were developed and it was thus possible to routinely obtain high-resolution diffraction (to better than 2.5 Å resolution). This novel and facile crystal-dehydration protocol may be useful for similar challenging situations.

## Abstract 2

The optimization of WbdD crystals using a novel dehydration protocol and experimental phasing at 3.5 Å resolution by cross-crystal averaging followed by molecular replacement of electron density into a non-isomorphous 3.0 Å resolution native data set are reported.

## 1. Introduction

The last two decades have seen a steady improvement of the infrastructure and techniques that are used to express, purify and crystallize proteins. Similarly, the actual structure-solution process has become increasingly streamlined (Winn *et al.*, 2011 ▶; Adams *et al.*, 2002 ▶; Winter, 2010 ▶). Structures can literally be solved by a single keystroke and the process is routine in many cases (Oke *et al.*, 2010 ▶). The remaining hurdle is obtaining reproducible high-quality crystals. This represents a particular problem for challenging targets such as protein complexes, membrane proteins and post-translationally modified eukaryotic proteins. Occasionally, however, even proteins that are anticipated to be routine prove to be difficult and their study can identify approaches for *a priori* challenging cases.

**Figure 1:**
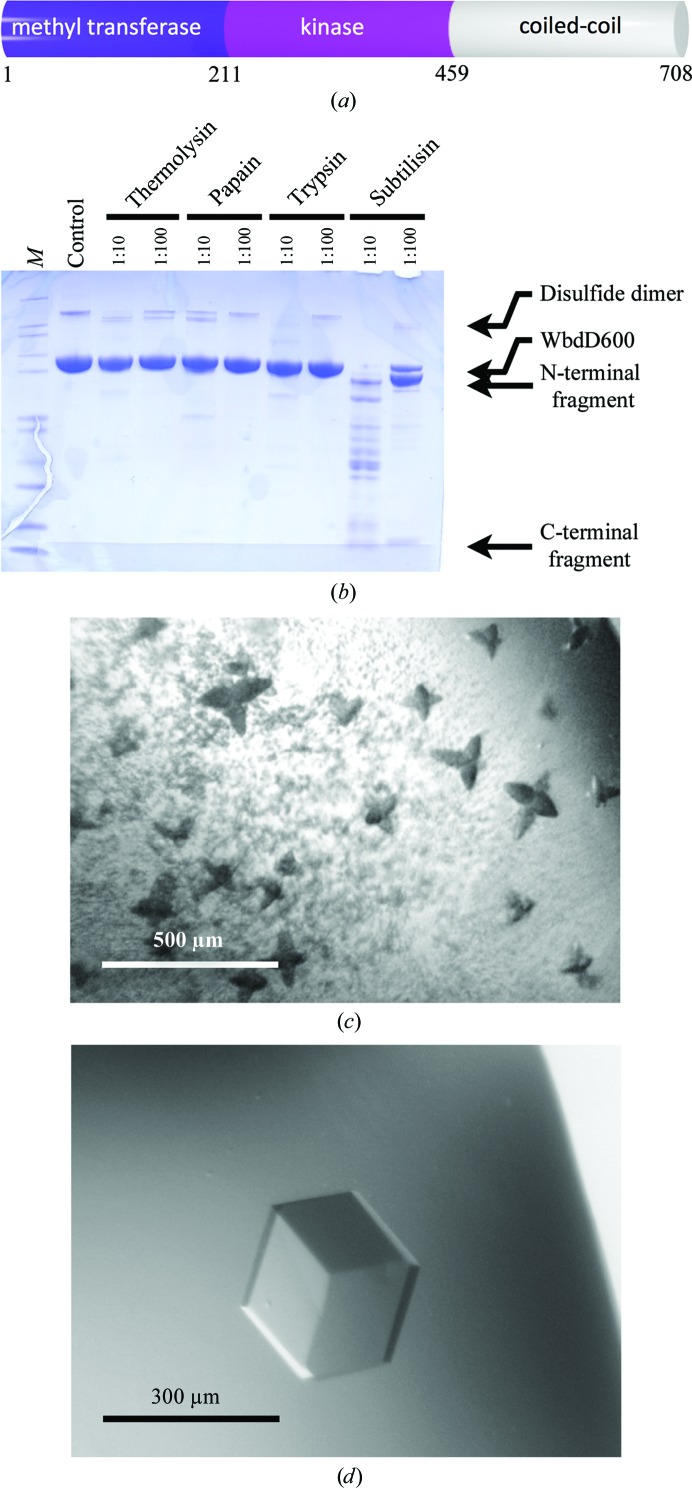
(*a*) The primary structure of WbdD. The domain borders were placed according to the crystal structure of WbdD556. (*b*) SDS–PAGE of WbdD600 samples from limited proteolysis reactions. Lane *M* contains NuPAGE Mark12 protein marker (Invitrogen). The molar ratio of protease:WbdD600 is indicated. (*c*) Initial WbdD556 crystals (see main text for the crystallization conditions). The dark colour of the crystals arises from the Izit stain (Hampton) that was used to confirm that the crystals are protein. (*d*) Optimized WbdD556 crystal.

WbdD is a soluble protein that controls the length of some lipopolysaccharide O-antigen polysaccharides that are synthesized in an ABC-transporter-dependent pathway (Cuthbertson *et al.*, 2010 ▶). WbdD from *Escherichia coli* O9a contains two enzymatic domains: a methyltransferase (MTase) domain and a kinase domain (Clarke *et al.*, 2004 ▶). WbdD stops polymerization of the chain by phosphorylating and then methylating the phosphate on the terminal sugar (Clarke *et al.*, 2009 ▶, 2011 ▶). The C-terminus of WbdD contains an amphipathic helix that locates WbdD at the cytoplasmic face of the inner membrane, as well as several predicted coiled-coil motifs (Fig. 1 ▶ *a*; Clarke *et al.*, 2004 ▶) in a region that interacts with the sugar polymerase WbdA (Clarke *et al.*, 2009 ▶), generating a complex for regulating chain extension and termination.

This bifunctionality of WbdD is unusual, so it was selected for study by X-ray crystallography. However, WbdD unexpectedly proved to be an exceptionally challenging case and here we report in detail how we tailored the expression construct, phased the structure experimentally at low resolution and finally improved the diffraction quality of our crystals by developing a new higher-throughput dehydration protocol for protein crystals.

## 2. Methods

### 2.1. Protein production

The nucleotide sequence encoding WbdD600 (residues 1–­600 of WbdD; accession No. JX235676) was cloned into pBAD24 as described previously (Clarke *et al.*, 2009 ▶). The same cloning strategy was used for the WbdD556 sequence. Both constructs introduced a tobacco etch virus (TEV) cleavable N-terminal His tag (MHHHHHHENLYFQG; only the C-terminal glycine remains as the new N-terminus after TEV cleavage, a one-residue extension of the sequence of the target protein). An identical expression and purification procedure was followed for both proteins. The plasmids were transformed into *Escherichia coli* Rosetta cells. A single colony was selected and grown overnight in Luria broth (LB) medium containing 100 µg ml^−1^ ampicillin. The overnight culture was used to inoculate 5 l LB medium at a ratio of 1:100. This culture was grown at 310 K with shaking at 200 rev min^−1^. Once the culture reached an optical density (600 nm) of 0.4, the incubation temperature was lowered to 301 K. At an OD_600_ of 1.0, protein expression was induced by adding l-­arabinose to a final concentration of 0.2% and the culture was incubated at 301 K for 5 h. The cells were harvested by centrifugation at 7000*g* for 30 min. Cell pellets containing the target proteins were resuspended in lysis buffer [20 m*M* bis-Tris, 250 m*M* NaCl, 10 m*M* imidazole, 5%(*w*/*v*) glycerol pH 7.0 and one Complete protease-inhibitor cocktail tablet (Roche Diagnostics) per 50 ml of extract] and the mixture was stirred at 277 K for 30 min. After treatment with a cell disrupter (207 MPa; Constant Cell Disruption Systems, Daventry, England), the lysate was clarified by centrifugation at 30 000*g* for 1 h at 277 K. The cell-free supernatant was loaded onto a 5 ml Ni–NTA column (GE Healthcare). Prior to loading, the resin was equilibrated in lysis buffer. The loaded resin was washed with lysis buffer and the target protein was eluted with lysis buffer containing 1 *M* imidazole. For final purification, the protein was passed over a Superdex 200 16/60 column (GE Healthcare) and eluted with 20 m*M* bis-Tris pH 7.0, 50 m*M* NaCl. Fractions with appropriate purity, as judged by SDS–PAGE, were pooled and concentrated to 10–20 mg ml^−1^ for crystallization. Protein identity and integrity were confirmed by mass spectrometry. The protein was flash-cooled in liquid N_2_ using thin-walled PCR tubes and stored at 193 K prior to further use.

### 2.2. Production of selenomethionine-labelled WbdD556

Selenomethionine-labelled WbdD556 was prepared using glucose-free SeMet medium from Molecular Dimensions. Glycerol was added at a concentration of 5% to provide a carbon source. The cells from a 100 ml overnight culture were harvested by centrifugation and resuspended in phosphate-buffered saline (PBS). The main culture was inoculated and grown for 1 h at 310 K before seleno-l-methionine (SeMet) was added (50 µg ml^−1^). When an OD_600_ of 0.5 was reached, the temperature was lowered to 301 K and protein expression was induced by adding 0.2% l-arabinose. The SeMet protein was purified in the same way as the native protein (see above).

### 2.3. Crystallization

Initial crystallization trials for WbdD600 and WbdD556 were performed using a Honeybee 963 robot system (Genomic Solutions) with both commercially available and self-made (Oke *et al.*, 2010 ▶) crystallization screens. For each of the 96-well sitting-drop vapour-diffusion screens (MRC plates, Swissci), 150 nl protein solution (∼15 mg ml^−1^ in 20 m*M* bis-Tris pH 7.0, 50 m*M* NaCl supplemented with 5 m*M* each of ATP, SAM and MgCl_2_) was mixed with 150 nl precipitant and equilibrated against a reservoir of 75 µl precipitant. The sealed plates were then incubated at 293 K.

Initial WbdD crystals were obtained in a condition containing 51% Tacsimate pH 8.0 from the commercial Index screen (Hampton Research) and were further optimized by several rounds of stochastic optimizations in a 96-well format. The best results, as judged by crystal appearance, were obtained with a mixture of 0.26 *M* lithium sulfate, 0.1 *M* Tris–HCl pH 8.4, 1.12 *M* ammonium sulfate. Crystals (>100 µm) usually appeared after 1–3 d at 293 K. Prior to flash-cooling in liquid nitrogen for data collection, the crystals were cryoprotected by transferring them into either a saturated ammonium sulfate solution or mother liquor supplemented with either 30% mannose, 30% ethylene glycol or 30% glycerol.

### 2.4. Limited proteolysis

The proteases thermolysin, papain, trypsin and subtilisin (Sigma) were mixed with WbdD600 (1 mg ml^−1^) in a 1:10 or a 1:100 molar ratio of protease:WbdD600. The mixtures were incubated on ice for 1 h and the reactions were stopped by adding boiling SDS–PAGE loading buffer (NuPAGE LDS sample buffer, Invitrogen). The samples were then analyzed by SDS–PAGE.

## 3. Results

### 3.1. Finding a suitable crystallization construct by limited proteolysis

WbdD is a 82 kDa protein with 708 amino-acid residues and two functional domains. The N-terminus of the protein contains a methyltransferase (MTase) domain (residues 1–210) followed by a kinase domain (residues 211–459) and a C-­terminal coiled-coil domain (residues 460–708) (Fig. 1 ▶ *a*). The full-length protein is difficult to solubilize and purify, but we found a construct comprising amino acids 1–600 of WbdD (WbdD600) that is expressed in sufficient amounts for crystallization experiments (>100 mg per litre of culture). We submitted the WbdD600 sequence to the *XtalPre*d server (Slabinski *et al.*, 2007 ▶) to assess the likelihood of crystallization and the protein was classified as ‘very difficult’. This is mainly owing to its relatively large size and the presence of multiple coiled-coil domains and the amphipathic helix (Fig. 1 ▶ *a*). Nevertheless, the protein was purified using a combination of Ni–NTA, ion-exchange and gel-filtration chromatography (Fig. 1 ▶ *b*, lane 2). Although more than 1000 crystallization conditions were tested at protein concentrations ranging from 5 to 40 mg ml^−1^, no crystals were observed. We subjected the protein to limited proteolysis in order to identify protease-resistant domains which might facilitate crystallization. Digestion with the protease subtilisin resulted in two stable cleavage products (Fig. 1 ▶ *b*). Analysis by mass spectrometry revealed that ∼50 amino acids were removed from the C-­terminus of the protein. We therefore designed, cloned and expressed a new construct, WbdD556 (residues 1–556), that was purified in the same way as WbdD600. Although this construct was still classified as ‘very difficult’ by *XtalPred*, it readily crystallized in a Tacsimate-based condition (51% Tacsimate pH 8.0, 5 m*M* SAM/Mg^2+^/ATP after optimization) based on the commercially available Index screen (condition 29; Hampton Research; Fig. 1 ▶ *c*). Condition 2.4 of The JCSG+ Suite (Molecular Dimensions) led to identical WbdD556 crystals growing from a mixture of lithium sulfate and ammonium sulfate (0.26 *M* lithium sulfate, 0.1 *M* Tris–HCl pH 8.4, 1.12 *M* ammonium sulfate, 5 m*M* SAM/Mg^2+^/ATP after optimization). We found that these crystals could also be obtained by *in situ* proteolysis (Dong *et al.*, 2007 ▶; Wernimont & Edwards, 2009 ▶) by crystallizing WbdD600 in the presence of low concentrations (1:1000 molar ratio of protease:WbdD) of subtilisin or trypsin.

### 3.2. WbdD crystals vary in diffraction quality

The initial optimization of the cubic (space group *I*23) WbdD crystals was based on their optical appearance and proved to be straightforward; large crystals (>200 µm) could be grown (Fig. 1 ▶ *d*). However, these crystals typically did not diffract to a resolution better than 7–8 Å (crystal category D). From over 500 tested crystals, only a dozen diffracted to 4–5 Å resolution (crystal category C) and only three crystals to around 3.5 Å resolution (crystal category B). An SeMet derivative gave similar results. During crystal screening, we noticed that the first crystal retrieved from a drop frequently diffracted significantly better than subsequently harvested crystals. We found a single native crystal that diffracted to better than 3.0 Å resolution (crystal category A), although there was no obvious visual indication as to why and this could not be reproduced. This crystal grew from the ammonium sulfate-based condition, but was harvested more than six months after the plate had been set up. During the intervening time, approximately half of the volume of the reservoir solution had evaporated, leaving a much higher (∼2 *M*) concentration of ammonium sulfate. By comparing the unit-cell parameters of the different crystal categories (A, B, C and D), we found that the decrease in length of the cubic unit-cell axis from 185 Å in crystal category D to 167 Å in crystal category A generally correlated with resolution. We interpreted this as an indication that dehydration of the WbdD556 crystals improved diffraction quality (Table 1 ▶).

### 3.3. Solving the selenium substructure and determining the correct hand

**Figure 2:**
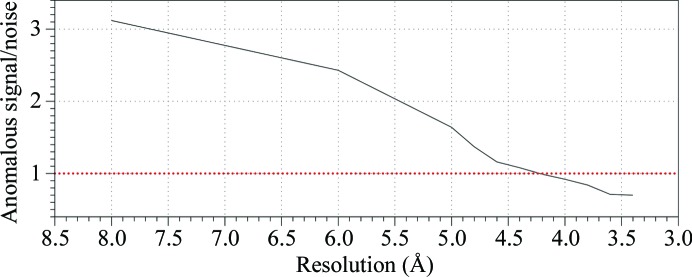
Strength 〈|*F* ^+^ − *F* ^−^|/[(σ_*F*+_)^2^ − (σ_*F*−_)^2^]^1/2^〉 of the anomalous signal *versus* resolution for the 3.5 Å resolution data set (Table 2 ▶) as calculated using *SHELXC* (Sheldrick, 2008 ▶). The red line at *y* = 0.8 indicates the threshold for the presence of an anomalous signal (Zwart, 2005 ▶).

**Figure 3:**
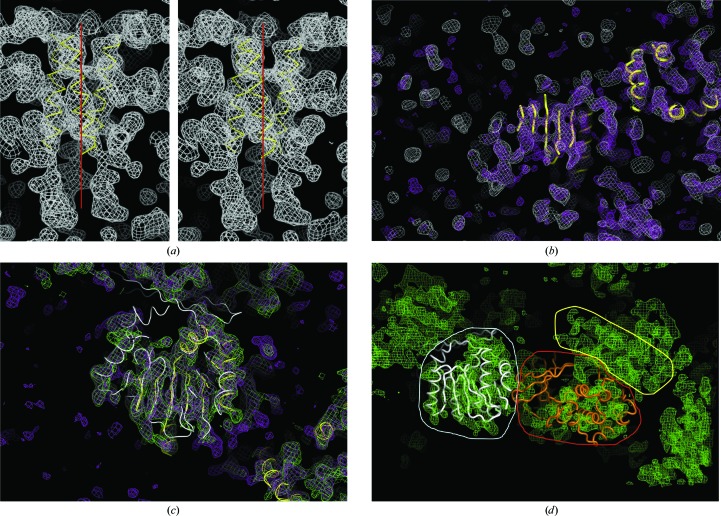
(*a*) Stereo pair of an electron-density map (1.0σ) calculated with the starting experimental phases after density improvement with *AUTOSOLVE* (Zwart *et al.*, 2008 ▶). The manually placed three-helix bundle is shown as a yellow tube model; the threefold crystallographic axis is indicated by a red line. (*b*) The white mesh represents the same map as shown in (*a*). The purple map was calculated from phases that were improved using *PARROT*. Manually placed secondary-structure elements are represented by yellow tubes. (*c*) The purple mesh is the same as in (*b*). The green map was calculated after cross-crystal averaging between the 3.5 Å resolution data set and the 4.2 Å resolution data set (Table 2 ▶) using *DMMULTI* (Winn *et al.*, 2011 ▶). Manually placed secondary-structure elements are represented by yellow tubes. The crystal structure (white tubes) of the SAM-dependent methyltransferase from *Pyrococcus horikoshii* OT3 (PDB entry 1wzn; RIKEN Structural Genomics/Proteomics Initiative, unpublished work) is superimposed onto the manually placed secondary-structure elements. (*d*) The green mesh is the same as in (*c*). The individual domains of WbdD556 are indicated (white, methyltransferase domain; red, kinase domain; yellow, three-helix bundle).

WbdD556 crystals were sensitive to radiation damage and the highest resolution SeMet data set was processed to 3.5 Å with *HKL*-2000 (Otwinowski & Minor, 1997 ▶; Table 2 ▶). The anomalous signal was found to extend to about 4.5 Å resolution using *SHELXC* (Sheldrick, 2008 ▶) and *phenix.xtriage* (Zwart *et al.*, 2008 ▶) (Fig. 2 ▶), and five of the expected seven selenium sites were located by *SHELXD* (Sheldrick, 2008 ▶). However, the phasing and density-improvement step in *SHELXE* (Sheldrick, 2008 ▶) did not result in an interpretable map or even clear solvent boundaries in either hand. We repeated the initial phasing steps with *phenix.hyss* (Grosse-Kunstleve & Adams, 2003 ▶; Zwart *et al.*, 2008 ▶) and* SOLVE*/*RESOLVE* (Terwilliger, 2003 ▶) from the *PHENIX* suite (Adams *et al.*, 2002 ▶), but obtained similar results. In the map corresponding to the original hand we noted a long tubular stretch of electron density wound around the crystallographic threefold axis, suggesting a coiled coil (Fig. 3 ▶ *a*). No similar structure was visible in the other hand. We inserted a 20-residue helix model using *Coot* and submitted the resulting crystallographic helical trimer to the *DALI* and *SSM* servers (Holm & Rosenström, 2010 ▶; Krissinel & Henrick, 2004 ▶). Both algorithms identified structures with similar arrangements of helices (*e.g.* PDB entries 1xkm, 2lfh and 3aha; Raimondo *et al.*, 2005 ▶; Northeast Structural Genomics Consortium, unpublished work; Izumi *et al.*, 2010 ▶), supporting the notion that the geometric arrangement of helices was reasonable. On this basis, we tentatively selected the hand.

### 3.4. Additional density improvement to build a crude model

The *Parrot* program (Winn *et al.*, 2011 ▶; Cowtan, 2010 ▶) improved the map quality (Fig. 3 ▶ *b*). The most significant difference was a better defined solvent boundary, which made it possible to identify the elongated shape of the molecule (Fig. 3 ▶ *b*). We also tentatively assigned secondary-structure elements. Our strategy at this point was to improve the model to a point where it would be possible to phase the 3.0 Å resolution native data set by molecular replacement. However this failed, indicating that the model was substantially incorrect. Owing to variations of the unit-cell length, we had a collection of non-isomorphous crystals (Tables 1 ▶ and 2 ▶) and we decided to try cross-crystal averaging with *DMMULTI*. We defined a mask around our crude model and cross-crystal averaged using the 3.0 Å resolution native data set, assuming that the mask occupied the same position relative to the threefold axis and allowing the matrix to refine. Unfortunately, the resulting maps were not interpretable. Employing the same procedure with a 4.2 Å resolution data set resulted in a much improved electron-density map (Fig. 3 ▶ *c*) and sufficient secondary-structural elements were fitted to confidently identify the MTase domain. This defined which part of the map corresponded to which of the two domains of WbdD. A model of Src kinase could be placed by hand into the remaining density, although the quality of fit was poor (Fig. 3 ▶ *d*).

### 3.5. Molecular replacement with electron density

**Figure 4:**
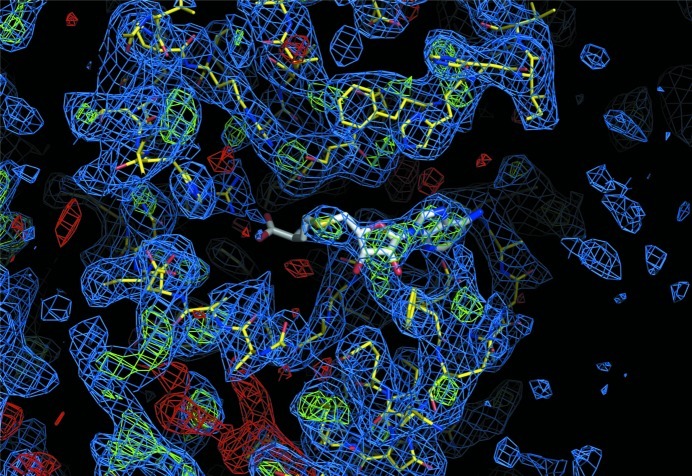
Electron-density map (blue, 2*m*|*F*_obs_| − *D*|*F*_calc_|, 1.0σ; green, *m*|*F*_obs_| − *D*|*F*_calc_|, 3.0σ; red, *m*|*F*_obs_| − *D*|*F*_calc_|, −3.0σ) calculated from phases directly after the masked electron densities shown in Fig. 3 ▶(*c*) were used as a model for molecular replacement in the 3.0 Å resolution data set (Table 2 ▶). The WbdD model (yellow) and the SAM cofactor (white) are shown as sticks.

**Figure 5:**
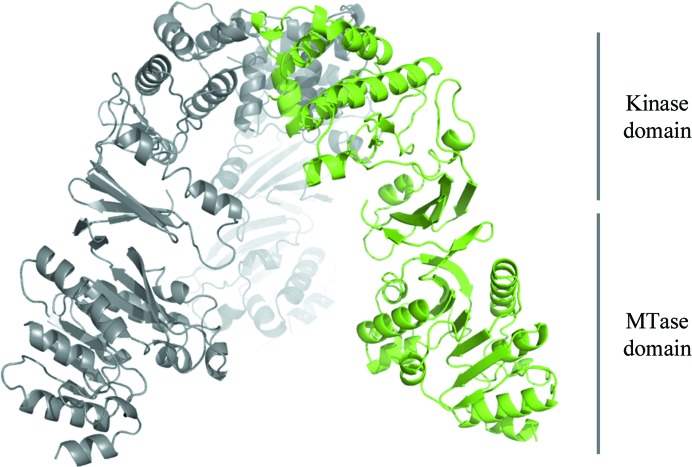
Cartoon model of the overall structure of a WbdD trimer. One monomer is coloured green and the other two are shown in grey. The relative positions of the MTase and kinase domains are indicated.

We suspected that the large changes in the unit-cell parameters were accompanied by domain reorganization within the crystal. We proceeded by using the separate domains for molecular replacement into the native 3.0 Å resolution data set and found a convincing solution for the MTase domain using *PHASER* (McCoy *et al.*, 2007 ▶; TFZ score 16.7, LLG 232), but no solution for the kinase domain was found. Inspection of the maps calculated from the molecular-replacement phases showed no additional difference electron density. This indicated that either the kinase domain was disordered in these crystals or our incorrect model biased the phases. We repeated molecular replacement using the density for each domain (rather than the structure) as the search model in an attempt to avoid model bias as much as possible. Once again, the MTase domain was confidently located by *PHASER* (McCoy *et al.*, 2007 ▶), but multiple borderline solutions were obtained for the kinase domain with low *Z*-scores of ∼6 and LLGs of around 40. Inspection of the Euler angles and translational shifts of the solutions revealed that the top solution of this set was in close proximity to the MTase domain solution. Phases calculated from the combined top solutions then resulted in electron density that was interpretable for both domains (Fig. 4 ▶). We were able to confidently build a complete medium-resolution model of WbdD and refine it by simulated annealing (*R* = 21.2%, *R*_free_ = 23.3%; Fig. 5 ▶, Table 3 ▶).

### 3.6. A new rapid dehydration method

Only after the preceding approaches resulted in a solved medium-resolution structure of WbdD were we able to develop a protocol that would give reproducible high-quality crystals for further study.

**Figure 6:**
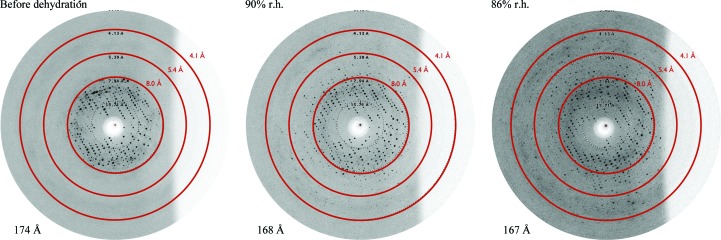
Right to left: dehydration of a WbdD556 crystal using a free-mounting system (Kiefersauer *et al.*, 2000 ▶). As indicated, the diffraction images were taken at different relative humidities (r.h.). The images were indexed with *HKL*-2000 (Otwinowski & Minor, 1997 ▶) to analyze the change in the cubic unit-cell parameter.

To investigate the role of crystal dehydration in determining data quality, we mounted crystals in a free-mounting system (FMS; Proteros; Kiefersauer *et al.*, 2000 ▶) and lowered the relative humidity (r.h.) of the gas phase surrounding the crystal. During this process, diffraction snapshots were taken and these revealed a clear improvement in the diffraction quality when the r.h. was lowered to ∼86% (Fig. 6 ▶). Indexing of the individual diffraction images revealed that in the initial stage (∼90% r.h.) the unit-cell parameter decreased (from 174 to 168 Å) and the diffraction quality improved (Fig. 6 ▶). Further dehydration (∼86% r.h.) led to only a slightly decreased unit-cell size but significantly improved the diffraction quality. The change in unit-cell parameters renders the crystals non-isomorphous. Below 86% r.h. the diffraction quality deteriorated and the process was not reversible. This is reminiscent of the complex dehydration behaviour observed in HIV-1 reverse transcriptase crystals (Esnouf *et al.*, 1998 ▶). WbdD556 crystals often cracked during dehydration with the FMS, degrading the diffraction quality. Covering the crystal in perfluoropolyether oil, setting the r.h. to 92% and allowing the dehydration to proceed overnight proved to be a reproducible procedure.

**Figure 7:**
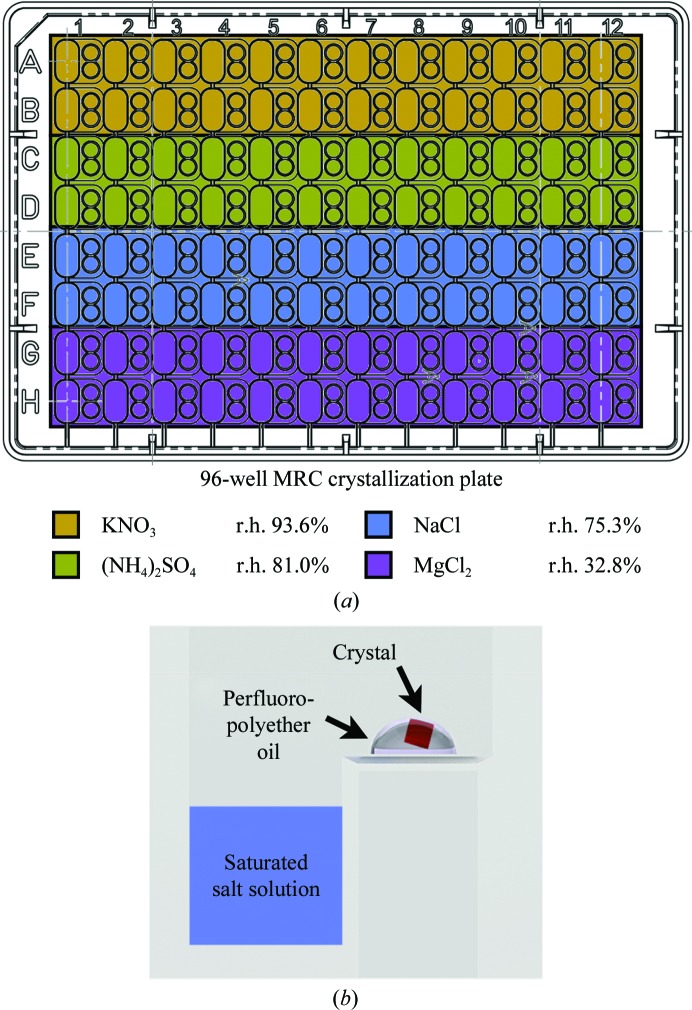
Schematic of our novel crystal-dehydration workflow. (*a*) A 96-well plate was filled with different saturated salt solutions. (*b*) The crystal (red) was harvested from its mother liquor and placed in a drop of perfluoro­polyether oil (PFO-X175/08; Hampton Research) on the sitting-drop shelf. The plate was then sealed and the setup was kept at room temperature for a week before the crystals were harvested and flash-cooled using liquid nitrogen prior to data collection.

From a practical perspective, however, this procedure was not suited for the production of multiple crystals. We therefore tested a commercially available dehydration kit (JBS Crystal Dehydration and Salvage Kit), in which the r.h. is controlled by a salt solution inside a cap which covers a mounted crystal. This was unsuccessful either because the salt often crystallized or because mechanical errors in placing the cap destroyed the crystal. A different approach was devised that involved filling the reservoirs of a 96-well crystallization plate (MRC plate) with different saturated salt solutions (*e.g.* potassium nitrate, ammonium sulfate, sodium chloride and magnesium chloride). Using a cryoloop (MiTeGen), crystals were then placed into 0.5 µl perfluoropolyether oil drops (one crystal per drop) in each well (Fig. 7 ▶). A cat whisker was used to push the crystal out of the cryoloop into the oil drop if required. The plate was then sealed and incubated for one week. Crystals were harvested from the oil drop and cryocooled in liquid nitrogen prior to the diffraction experiment. With WbdD, dehydration with ammonium sulfate (r.h. 81%) reproducibly yielded crystals that diffracted to better than 2.5 Å resolution. The best diffracting crystals (which also belonged to space group *I*23) had a unit-cell parameter of 158 Å, which is again considerably shorter than the 167 Å unit-cell parameter of the native crystal that was used to solve the initial structure (Table 2 ▶). The resulting high-resolution structure, complexes, mutants and biological implications will be described elsewhere.

## 4. Discussion

### 4.1. Effect of dehydration on the structure

**Figure 8:**
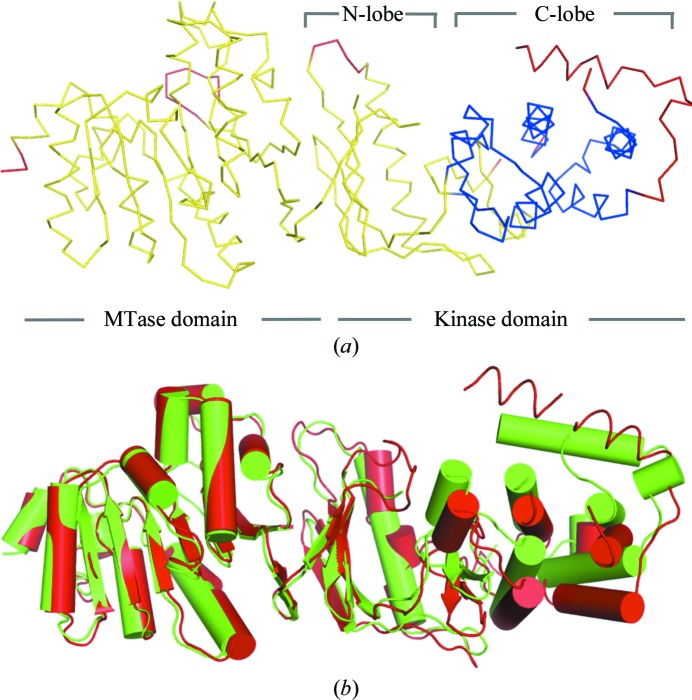
(*a*) Rigid bodies (yellow and blue ribbons) in WbdD556 identified by the *RAPIDO* webserver (Mosca & Schneider, 2008 ▶). Hinge regions are shown as red ribbons and the cofactors ATP and SAM as spheres. (*b*) Superposition of the 3.0 Å resolution (red) and 4.2 Å resolution (green) WbdD structures (Table 2 ▶) showing the twisting motion that occurs during crystal dehydration and pivots around the ATP-binding cleft. The superposition is based on the rigid body that comprises the methyltransferase and the N-lobe of the kinase domain [coloured yellow in (*a*)].

**Figure 9:**
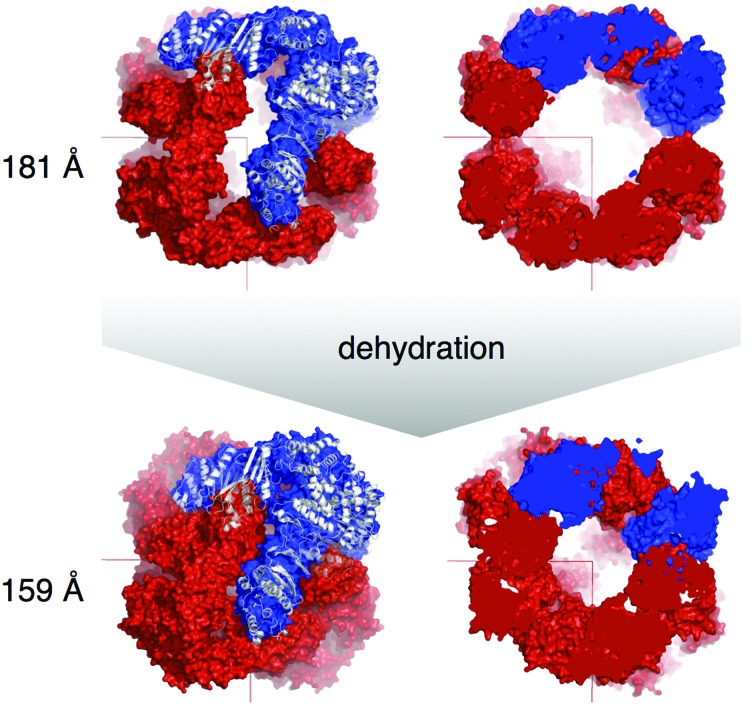
Effect of dehydration on the trimeric WbdD structure and the crystal packing. The top row shows the packing of the non-dehydrated crystal and the bottom row that of the dehydrated crystal. One of the four WbdD556 trimers (surface representation) which are centred around each unit-cell corner is shown in blue with a superimposed cartoon representation of the protein (white). In the figure at the top right, the pyramid-shaped assembly shown on the left is cut open to reveal the size of the internal hollow space.

After the structure of WbdD556 had been solved as described above, the model was used to solve the structure of the 4.2 Å low-resolution data set (Table 2 ▶) in order to investigate the structural changes that occurred during dehydration. The *RAPIDO* webserver was employed to align the two structures (Mosca & Schneider, 2008 ▶; Mosca *et al.*, 2008 ▶). The algorithm detected two rigid bodies in WbdD. The first one comprises residues 7–194, 203–230, 239–259, 264–314, 340–351 and 368–376 and represents the MTase domain together with the N-­lobe of the kinase domain (Fig. 8 ▶). The second rigid body contains the C-lobe of the kinase domain (residues 315–339, 352–367, 383–398 and 418–449). Superposing both structures based on the first rigid body revealed a twisting motion with a hinge between the N- and C-lobes of the kinase domain (ATP-binding cleft). In the highest resolution (better than 2.5 Å) WbdD structures the ATP added to the crystallization conditions was disordered. In these structures, the twisting motion displaces the nucleotide from the binding pocket by inserting protein residues into the binding cleft. There is a 20° movement of the C-terminal helix that forms the three-helix bundle in the trimer. In the high-resolution structure this helix is in much closer contact with the kinase domain (Fig. 8 ▶). In retrospect, the rigid-body motion within the kinase domain probably explains why molecular replacement based on a single kinase domain failed. The structural changes are striking when viewed in the crystal-packing environment (Fig. 9 ▶). Four WbdD trimers organize themselves into a pyramid-shaped arrangement that is centred on each corner of a unit cell. During dehydration, this assembly is dramatically compressed as the three arms of the trimers bind to each other. This may explain why dehydration is so reproducible in yielding good-quality crystals (Fig. 9 ▶).

We analyzed the two crystal-packing arrangements (dehydrated and non-dehydrated) using the *PISA* server (Krissinel & Henrick, 2007 ▶). In the non-dehydrated form each WbdD556 monomer with a surface area of ∼21 000 Å^2^ contributes to three protein–protein interfaces. Each of these has a relatively small interface area of ∼200 Å^2^ formed by 6–8 amino-acid residues. After dehydration, each WbdD556 monomer is involved in five protein–protein interfaces; four of these have interface areas between 400 and 700 Å^2^ and involve up to 26 amino-acid residues at each interface. In the dehydrated state, the interactions between WbdD556 monomers are so extensive that *PISA* classifies both the whole pyramid-shaped assembly (Fig. 9 ▶) and the trimer as being stable in solution. In contrast, *PISA* did not define any of the interfaces in the ‘non-dehydrated’ packing as being stable in solution. This included the trimer, which can be identified in solution by gel filtration (data not shown).

Each of the WbdD556 structures contains an ∼20-residue α-­helix remaining from the C-terminal coiled-coil domain (Fig. 1 ▶ *a*). In the lower-resolution structures we could model 5–6 additional residues of the helix, but the remaining ∼80 residues were always disordered. For four WbdD556 trimers in one pyramid (Fig. 9 ▶), almost 1000 amino acids would have to fit into the central cavity. A simple volume calculation revealed that the void is indeed large enough to accommodate the missing residues unless the remaining residues formed a single helix. The volume of the cavity shrinks during dehydration, perhaps explaining why in contrast to the rest of the structure the helical bundle becomes more disordered as the crystals are dehydrated. The void is too small for constructs that have a longer C-terminal bundle than WbdD556 (Fig. 9 ▶).

More than ten years ago, at the outset of structural genomics (SG) programs, our laboratory solved the structure of UDP-galactopyranose mutase (Sanders *et al.*, 2001 ▶). This project represented a challenge owing to varying crystal quality and non-isomorphism between phased and unphased diffraction data sets. Despite the transformation in infrastructures (*e.g.* beamlines) and software packages in the intervening years, very similar problems were encountered with WbdD. One key element in the process of solving the structure of WbdD was the discovery of a plausible three-helix bundle in one low-resolution electron-density map (Fig. 3 ▶ *a*). The quantity of the structural data in the PDB made this possible. Density-modification software such as *DMMULTI* (Winn *et al.*, 2011 ▶) and *PARROT* (Cowtan, 2010 ▶) was essential in solving the structure of WbdD. In a counter-intuitive outcome, dramatically improved electron density was achieved by cross-crystal averaging with the non-isomorphous low-resolution (but not the high-resolution) data set (4.2 Å resolution; Table 2 ▶; Fig. 3 ▶ *c*). Use of electron density as a molecular-replacement model with, for example, *PHASER* (McCoy *et al.*, 2007 ▶) is now much more automated and this was also critical to our success. Although multiple cases are known in which crystals were successfully dehydrated to improve their diffraction properties (see, for example, Esnouf *et al.*, 1998 ▶; Kiefersauer *et al.*, 2000 ▶; Chotiyarnwong *et al.*, 2007 ▶), it is still a technique that is rarely reported. This perhaps reflects frequent failure or technical difficulties. WbdD may be a special case because the high symmetry (*I*23) of the crystal lattice leads to isotropic changes in the crystal and the novel packing arrangement was particularly favourable (Fig. 9 ▶). The effect of dehydration on WbdD (an improvement in diffraction resolution from 8 to 2.2 Å) is very pronounced when compared with other examples (Heras & Martin, 2005 ▶). However, a facile and easily implementable protocol may extend the utility of dehydration. The procedure described here combines several previously published ideas (Kiefersauer *et al.*, 2000 ▶; Heras & Martin, 2005 ▶) into an easy-to-use, low-cost and convenient workflow. It is not anticipated that this will necessarily lead to more success than other approaches, but it is straightforward and amenable to high throughput. With robotic mounting and testing of samples, the technique may be appropriate for routine application for poorly diffracting high-solvent crystals.

## Figures and Tables

**Table 1:** Overview of WbdD556 crystals that had been tested and/or used for data collection at the time when the structure was solved

Resolution (Å)	No. of crystals	Unit-cell parameter (Å)	Type of crystal
7–8	>500	∼185	Native, SeMet
4–5	∼20	∼182	Native, SeMet
3.5–4	3	∼177	SeMet
3.0	1	167	Native

**Table 2:** Data-collection statistics for WbdD556 The 3.0 Å resolution data set was processed so that all data with *I*/σ(*I*) > 1.0 were included. Values in parentheses are for the highest resolution shell

	3.0 Å, native	3.5 Å, SeMet	4.2 Å, native
Space group	*I*23	*I*23	*I*23
Unit-cell parameters (Å, °)	*a* = *b* = *c* = 167.2, α = β = γ = 90	*a* = *b* = *c* = 177.6, α = β = γ = 90	*a* = *b* = *c* = 180.0, α = β = γ = 90
Matthews coefficient (Å^3^ Da^−1^)	3.1	3.7	3.88
Solvent content (%)	61	67	69
Molecules per asymmetric unit	1	1	1
Resolution range (Å)	30.0–2.80 (2.85–2.80)	55.0–3.52 (3.59–3.52)	63.6–4.22 (4.33–4.22)
Total observations	197495	81347	153562
Unique reflections	19185	11716	7119
Completeness (%)	99.5 (97.5)	95.6 (98.3)	100 (100)
*R*_merge_ † (%)	10.3 (200)	8 (84)	20 (57.1)
Multiplicity	10.3	6.9	21.6
〈*I*/σ(*I*)〉	20.1 (1.1)	30.2 (2.2)	14.8 (6.0)

† *R*_merge_ = 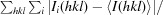 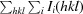, where *I*_*i*_(*hkl*) is the intensity for all observations *i* of reflection *hkl* and 〈*I*(*hkl*)〉 is the weighted average intensity for all observations *i* of reflection *hkl*.

**Table 3:** Refinement statistics for WbdD556 The structure has been deposited in the PDB as entry 4ax8.

	3.0 Å, native
*R* (%)	21.2
*R*_free_ (%)	23.3
R.m.s.d. bonds (Å)	0.017
R.m.s.d. angles (°)	1.835
Ramachandran plot (%)	
Allowed	97.3
Disallowed	0.2
*MolProbity* † score	1.86
*MolProbity* † clashscore	7.01

† Chen *et al.* (2010 ▶).
